# Adherence to the MIND diet is inversely associated with odds and severity of anxiety disorders: a case–control study

**DOI:** 10.1186/s12888-023-04776-y

**Published:** 2023-05-10

**Authors:** Kimia Torabynasab, Hossein Shahinfar, Shima Jazayeri, Mohammad Effatpanah, Leila Azadbakht, Jamileh Abolghasemi

**Affiliations:** 1 Department of Nutrition, School of Public Health, Iran University of Medical Sciences, Tehran, Iran; 2 School of Medicine, Imam Khomeini Hospital, Tehran University of Medical Sciences, Tehran, Iran; 3 Department of Community Nutrition, School of Nutritional Sciences and Dietetics, Tehran University of Medical Sciences, Tehran University of Medical Sciences, Tehran, Iran; 4 Department of Biostatistics, Iran University of Medical Sciences, Tehran, Iran

**Keywords:** MIND diet, Anxiety disorders, Psychiatric disorders, Mediterranean diet, DASH

## Abstract

**Background:**

The association between the Mediterranean-DASH diet Intervention for Neurodegenerative Delay (MIND) diet, odds, and severity of anxiety disorders (AD) is still unclear. We aimed to investigate whether adherence to MIND diet is associated with odds and severity of AD.

**Methods:**

The present case–control study carried out on 85 patients who were group matched by gender with 170 healthy subjects. Data for dietary intake was assessed by using a 147-item validated food frequency questionnaire (FFQ). Anthropometric measures were collected using standard methods. The MIND diet score was calculated using FFQ. We assessed anxiety disorder severity using the Generalized Anxiety Disorder-7 (GAD-7) questionnaire. Multivariate odds ratios (OR) with 95% confidence intervals (CI) were used to investigate the association of MIND diet and anxiety disorder.

**Results:**

We observed that higher adherence to MIND diet was associated with the lower GAD-7 score (*p* < 0.001). Individuals in the top category of MIND diet score were 97% less likely to have AD compared with those in the bottom category (OR: 0.03, 95% CI: 0.01, 0.09). There was significant reverse linear association between MIND diet score and AD (β = -3.63, *p* < 0.001).

**Conclusions:**

In conclusion, we provided some evidence indicating negative association between adherence to MIND diet, odds, and severity of AD. Finally, due to the probable preventive role of diet, it is vital to clarify the association between diet and AD through large-scale prospective cohort studies in the future.

## Introduction

Anxiety disorders (AD) are one of the most common psychological disorders [1]. Approximately one in four people meets the diagnostic criteria for at least one anxiety disorder based on the national comorbidity study with a prevalence rate of 17.7 percent over a 12-month period [2]. In Iran, the annual prevalence of anxiety disorders for male and female was 12.0% and 15.6% respectively with total prevalence of 15.6% [3].

Diagnostic and Statistical Manual of Mental Disorders, 5th edition (DSM-5) criteria are used in diagnostic process of anxiety disorders taking the type, number and duration of symptoms into account in accordance with each disorder specific symptom [2]. Main symptoms of anxiety disorders like increased heart rate, chest pain and sweating are attributed to amplified activation of sympathetic and noradrenergic systems [4]. Also, individuals with anxiety disorders experience significant and uncontrollable feelings of anxiety and fear, affecting wide spectrum of daily life from personal function to social communications [4].

A growing body of research has focused on how anxiety disorders impact the quality of life. According to epidemiological surveys, clinical studies, and qualitative literature reviews, researchers have found that patients with anxiety disorder experience substantial impairments in quality of life (QOL), comparable to that observed in other psychiatric conditions [5, 6]. Besides, results of a meta-analysis comparing anxiety disorder patients with nonclinical controls indicated that across multiple comparisons, using various QOL measures, anxiety patients had a poorer overall quality of life than controls with a large effect size [7].

It is well-established that high-quality diets are associated with great protection against psychological health problems, which is attributed to the anti- and pro-inflammatory properties of food and dietary components [8]. As such, preventing psychological issues by addressing modifiable risk factors is of high importance. Previous published papers have illustrated the beneficial effects of DASH and Mediterranean diet on mental wellbeing [9–12]. The Mediterranean-DASH diet Intervention for Neurodegenerative Delay (MIND) diet is a newly developed dietary pattern playing neuroprotective roles and is associated with cognitive and age-related neurodegenerative impairment alleviations [13–16]. It is developed by integration of the Mediterranean diet and Dietary Approaches to Stop Hypertension (DASH) [15]. MIND diet accentuates plant-based foods, while limiting high saturated fat-containing and animal foods [15, 17]. This dietary pattern classifies green leafy vegetables, berries, and pastries in separate categories reflecting modifications related to neuroprotection [15]. Some previous studies have been conducted on the relationship between the MIND diet and psychological issues such as depression and anxiety symptoms [18–20]. However, data is limited and results are controversial. MIND diet is enriched with bioactive compounds such as polyphenols, carotenoids, vitamin E, C, and B vitamins, which are essential for the proper function of neurons [21]. Currently, no studies have been conducted on MIND diet and anxiety disorders, but previous studies have looked at diet quality and nutrient intake as related to anxiety disorders [22–26]. Additionally, the association between anxiety symptoms and adherence to MIND diet was investigated in one study showing no significant association between consumption of MIND diet and odds of anxiety symptoms [18]. However, no study has been conducted on anxiety disorders with structured interview for anxiety diagnosis. Thus, the purpose of this study was to investigate the relationship between adherence to the MIND diet in adults with anxiety disorders whose disorder was diagnosed by the physiatrist.

## Methods

### Study design and population

This case–control study was carried out between 2021 and 2022 on two groups of patients suffering from anxiety disorders (*n* = 85) and apparently healthy people (*n* = 85) in Imam Khomeini hospital and referral psychology clinics. To select the subjects, convenience sampling was applied. A total sample size of 170 individuals was calculated using data from study and using with 80% power and 95% confidence interval ($n=\frac{{\left({Z}_{1-{}^{\alpha }\!\left/ \!{}_{2}\right.}+{Z}_{1-\beta }\right)}^{2}{\sigma }^{2}}{{\left({\mu }_{1}-{\mu }_{2}\right)}^{2}})$, α = 0.05, β = 0.20) [27]. Participants were group matched by gender. Increasing the power of the study, however, we continued sampling until the control group had twice as many participants as the case group. Ultimately, a total of 255 participants took part in our study. Diagnosis procedure and questionnaires fulfillment were performed during the consultation session. People who have been diagnosed with an anxiety disorder by a psychiatrist using DSM-5 criteria, created the patient group. On the other hand, people who came to the psychology clinic for diverse counseling courses except psychological disorders, and did not meet anxiety disorders criteria based on DSM-5 created control group. Inclusion criteria were considered for both groups of participants in our study, such as not taking anti-anxiety or anti-depression medications within the last 2–3 months, being between the ages of 18 and 60, having a body mass index (BMI) between 18.5 and 30, and to be apparently healthy. Apparently healthy adults are those who were not diagnosed as having any disease according to their statement. Those who did not answer more than 80% of the questionnaire questions, and had consumed less than 800 or more than 4200 cal per day were excluded [28].

### Demographic data collection

Using self-administered questionnaires, participants’ information including gender, age, health status, education, marital status, supplements, and medicine consumption, and smoking were all collected. We used a short, valid and reliable version of the International Physical Activity Questionnaire (IPAQ) to evaluate the level of physical activity of each participant in the past 7 days [29]. Using IPAQ, the evaluation of vigorous or moderate physical activities, walking, and sitting time of the subjects was performed. Then, to estimate the total weekly physical activity of each participant, the Metabolic Equivalents (METs) were computed as (MET-min/week). Ultimately, subjects were classified into three categories: very low (below 600 MET-minutes/week), low (600–3000 MET-minutes/week), moderate, and high (above 3000 MET-minutes/week) [30].

### Anthropometric data collection

Weight measurement was conducted by Digital scale (Seca 707; Seca GmbH & Co. KG., Hamburg, Germany), while participants were minimally dressed without shoes, and a using a stadiometer (Seca, Germany), height was measured in a standing position without shoes to the nearest 0.5 cm. The BMI was calculated by dividing the weight (kg) by the square of the height (m2).

### Dietary intake assessment

We estimated participants' dietary intake by validated semi-quantitative 147-item food frequency questionnaire designed based on Iranian foods [31]. For each food item, participants answered two questions: How often were food groups consumed (annually, monthly, weekly, and daily in the past year); and approximately how much were consumed each time? Following that, the amount and frequency of consumption of all food items were converted to grams per day using "household measures" [32]. Macronutrients and micronutrients content of diets were calculated using modified Nutritionist IV software developed for Iranian foods (version 7.0; N-Squared Computing, Salem, OR, USA.

### Calculation of adherence to the MIND diet

MIND diet score is computable by FFQ-derived data. According to the original MIND diet scoring, it consists 15 dietary groups, classified as brain-healthy or brain-unhealthy food groups. 10 food groups including green leafy vegetables, other vegetables, nuts, berries, beans, whole grains, fish, poultry, olive oil, and wine are in brain healthy category, while 5 food groups including red meats, butter and margarine, cheese, pastries and sweets, and fast food are in brain-unhealthy category [33].

In the current study, wine was not included in the MIND diet due to the lack of information in the FFQs; therefore, the consumption of wine was not considered in the scoring process, and MIND score was constructed using the remained 14 components. Constructing MIND score, individuals were categorized into tertiles based on how much of these 14 food groups they consumed. Participants in the lowest tertile received a score of 0, those in the middle a score of 0.5, and those in the top tertile received a score of 1. Regarding harmful food groups, the scoring was reversed; participants in the lowest tertile received scores of 1, those in the middle tertile received scores of 0.5, and those in the highest tertile received scores of 0. The scores for all the food groups were summed up to create the final MIND diet score. As a result, every individual has a score between 0 and 14.

### Assessment of anxiety disorder

The DSM-5 criteria were used by the psychiatrist in our study to diagnose anxiety disorder. We assessed anxiety disorder severity using the GAD-7 questionnaire that is specifically developed for anxiety disorders assessment and also has demonstrated adequate psychometric properties and diagnostic accuracy in the Iranian population [34]. The GAD-7 cut points of 5, 10, and 15 correspond to mild, moderate, and severe anxiety, respectively [35].

### Statistical analysis

Statistical tests such as Kolmogorov–Smirnov, Shapiro–Wilk and the Q-Q plot were used to determine the normality of distributions. After that, participants were categorized based on the tertiles of MIND scores. The independent sample t-test was used to evaluate the differences between the two groups. We used one-way analysis of variance (ANOVA) for quantitative variables and chi-square tests for qualitative variables to compare general characteristics among tertiles of the MIND score. Data were shown as the mean ± SD for continuous variables and percent (%) for categorical ones. Dietary intakes were energy-adjusted by the residual model [36]. To compare energy-adjusted dietary intakes of subjects across tertile of MIND, analysis of covariance (ANCOVA) was applied. We used Analysis of covariance (ANCOVA) to compare GAD-7 score among tertiles of MIND (adjusted for age, sex, energy intake, marital status, education, medicine use, vitamin supplements use, smoking status, alcohol consumption, physical activity, health status, and past medical history, and BMI). Multiple linear regression analysis was used to evaluate the association between AD and MIND score after adjustment for covariates, including age, sex, energy intake, marital status, education, medicine use, vitamin supplements use, smoking status, alcohol consumption, physical activity, health status, and health past medical history, and BMI. We used multinomial logistic regression analysis in different models. First, we controlled for the confounding effect of age, sex, and energy intake. In the second model, we further adjusted for marital status, education, medicine use, vitamin supplements use, smoking status, alcohol consumption, physical activity, health status, and past medical history. In the final model, we additionally adjusted for BMI. All statistical analyses were performed using The Statistical Package for the Social Sciences (SPSS version 25; SPSS Inc.) We considered *p* < 0.05 as significance level.

## Results

General characteristics of study participants between cases and controls are presented in Table 1. There were no statistically significant differences in age, weight, height, BMI, sex, marital status, education status, alcohol intake, smoking, dietary supplement use, health status, medication use and physical activity level between the case and control groups.

**Table 1:** General characteristics of patients with anxiety disorders and controls

	**control** **(170)**	**case** **(85)**	*P*-value*
Age (year)	33.61 ± 11.23	35.52 ± 12.74	0.220
Weight (kg)	69.31 ± 12.92	68.11 ± 13.36	0.482
Height (cm)	166.21 ± 12.91	165.54 ± 8.85	0.683
BMI (kg/m2)	25.71 ± 12.94	24.72 ± 4.25	0.461
Gender, n (%)			0.623
Male	49 (64.56)	27 (35.44)	
Female	121 (67.62)	58 (32.38)	
Marital Status, n (%)			0.281
Married	80 (63.56)	46 (36.44)	
Single	90 (69.87)	39 (30.13)	
Education Status, n (%)			0.373
Undergraduate	133 (68.91)	60 (31.09)	
Diploma	6 (54.54)	5 (45.46)	
College education	31 (60.84)	20 (39.16)	
Alcohol, n (%)			0.745
Yes	14 (70.01)	6 (29.99)	
No	156 (66.45)	79 (33.55)	
Smoke, n (%)			0.182
Yes	23 (57.52)	17 (42.48)	
No	147 (68.47)	68 (31.53)	
Health status n (%)			0.501
Healthy	156 (66.15)	80 (33.95)	
Non-healthy	14 (73.71)	5 (26.29)	
Past medical history n (%)			
Healthy	160 (68.14)	75 (31.86)	0.101
Non-healthy	10 (50.04)	10 (49.96)	
Dietary supplement use, n (%)			0.083
Yes	105 (70.92)	43 (29.08)	
No	65 (60.74)	42 (39.36)	
Medication use, n (%)			0.192
Yes	21 (77.85)	6 (22.15)	
No	149 (65.46)	79 (34.54)	
Activity level, n (%)			0.281
Low	67 (61.53)	42 (38.47)	
Moderate	64 (71.98)	25 (28.02)	
High	39 (68.45)	18 (31.55)	

Values are based on mean ± standard deviation or reported percentage

Chi-2 test for categorical variables and Student *t* test for continuous variables have been used

*BMI* body mass index, *Kg/m*^*2*^ kilogram/meter, *cm* centimeter, *n* number

^*^P value less than 0.05 was considered significant

General characteristics of study participants according to tertiles of MIND score are presented in Table 2. Compared to those in the lowest tertile, subjects in the top tertile were younger, had higher height, and had lower BMI. Distribution of a current health status, past medical history, medication use, supplement use, marital status, education status and smoking were different across tertiles of MIND score. There was no significant difference in distribution of sex, alcohol use, and physical activity of participants across tertiles of MIND score. There was no significant difference in weight across tertiles of MIND, as well.

**Table 2:** General characteristics of the participants in the study across tertiles of MIND

	**MIND**			***P* value**
	**T1** **85**	**T2** **74**	**T3** **96**	
n				
MIND score range	(1.5,6.5)	(7,9)	(9.5–13)	
Age (year)	39.71 ± 12.73	31.62 ± 9.09	31.33 ± 11.02	< 0.001
Weight (Kg)	69.71 ± 12.72	67.73 ± 12.71	69.01 ± 13.62	0.622
Height (m)	162.11 ± 14.34	168.21 ± 8.96	168.14 ± 10.15	0.001
BMI (kg/m^2^)	27.91 ± 17.63	23.72 ± 3.56	24.42 ± 4.49	0.029
Sex, n (%)				
Male	22 (28.93)	20 (26.32)	34 (44.75)	0.310
Female	63 (35.24)	54 (30.12)	62 (34.64)	
Current health status n (%)				
Healthy	74 (31.42)	72 (30.44)	90 (38.14)	0.042
Non-healthy	11 (57.96)	2 (10.41)	6 (31.63)	
Past medical history n (%)				
Healthy	72 (30.62)	68 (28.92)	95 (40.46)	0.002
Non-healthy	13 (65.04)	6 (30.03)	1 (4.93)	
Medication n (%)				
Yes	17 (63.05)	4 (14.74)	6 (22.21)	0.003
No	68 (29.73)	70 (30.71)	90 (39.56)	
Supplement n (%)				
Yes	53 (35.82)	32 (21.53)	63 (42.65)	0.008
No	32 (29.80)	42 (39.37)	33 (30.83)	
Marital status n (%)				
Single	63 (50.00)	30 (23.80)	33 (26.20)	< 0.001
Married	22 (17.10)	44 (34.10)	63 (48.80)	
Education n (%)				
Undergraduate	7 (63.64)	4 (36.36)	0 (0.00)	0.006
Diploma	23 (45.14)	15 (29.41)	13 (25.45)	
College education	55 (28.51)	55 (28.51)	83 (42.98)	
Smoking, n (%)				
Yes	13 (32.52)	18 (45.05)	9 (22.43)	0.029
No	72 (33.54)	56 (25.90)	87 (40.56)	
Alcohol, n (%)				
Yes	9 (45.00)	11 (55.00)	0 (0.00)	0.494
No	76 (32.34)	63 (26.75)	96 (40.91)	
Physical activity, n (%)				
Low	40 (36.71)	29 (26.58)	40 (36.71)	0.303
Moderate	27 (30.32)	23 (25.85)	39 (43.83)	
High	85 (33.36)	74 (29.04)	96 (37.60)	

*P* value less than 0.05 was considered significant

Values are based on mean ± standard deviation or reported percentage

One-way analysis of variance (ANOVA) for quantitative data and Chi-2 test for qualitative data have been used

*MIND* Mediterranean-DASH diet Intervention for Neurodegenerative Delay, *BMI* body mass index, *cm* centimeter, *kg* kilogram, *m* meter, *n* numbers

Subjects in the first tertile of MIND had MIND score between (1.5,6.5); second tertile: between (7,9); third tertile: between (9.5–13)

Food groups according to the tertiles of the MIND diet were indicated in Table 3. Individuals in the third tertile of MIND diet had higher intake of green leafy vegetables (*p* < 0.001), other vegetables (*p* < 0.001), whole grains (*p* = 0.01), berries (*p* < 0.001), fish (*p* = 0.004), beans (*p* < 0.001), poultry (*p* < 0.001) as well as lower intake of pastries (*p* < 0.001), and fast foods (*p* < 0.001), butter and margarine (*p* = 0.03), and red meat (*p* = 0.003). The intake of other food groups was not significantly different across tertiles of MIND diet.

**Table 3:** Dietary intakes of the study participants according to Tertiles of MIND score

Tertiles of MIND				
	T1	**T2**	T3	***P*-value**
**MIND score ranges**	(1.5,6.5)	(7,9)	(9.5,13)	
**Subjects, n**	85	74	96	
**Green leafy vegetables, g/d**	19.32 ± 10.13	59.72 ± 117.01	49.65 ± 30.21	< 0.001
**Other vegetables, g/d**	120.51 ± 70.73	232.45 ± 121.11	275.41 ± 108.21	< 0.001
**Whole grains, g/d**	61.03 ± 119.51	48.22 ± 51.93	86.14 ± 71.15	0.010
**Berries, g/d**	2.65 ± 10.67	6.16 ± 9.06	8.97 ± 8.74	< 0.001
**Olive oil, g/d**	0.49 ± 2.78	2.22 ± 10.7	2.19 ± 4.27	0.140
**Nuts, g/d**	7.32 ± 15.07	9.68 ± 16.51	10.82 ± 7.83	0.190
**Fish, g/d**	7.63 ± 45.03	9.16 ± 12.22	20.91 ± 19.37	0.004
**Beans, g/d**	6.44 ± 15.02	26.01 ± 28.53	27.62 ± 19.54	< 0.001
**Butter and margarine, g/d**	6.05 ± 13.91	4.29 ± 18.01	1.17 ± 2.11	0.030
**Cheese, g/d**	23.35 ± 46.38	19.23 ± 17.03	18.65 ± 14.72	0.500
**Red meat, g/d**	65.21 ± 115.09	32.75 ± 32.42	31.62 ± 29.61	0.003
**Fast foods, g/d**	37.03 ± 85.32	9.63 ± 11.56	10.47 ± 10.23	< 0.001
**Pastries, g/d**	132.44 ± 126.02	71.65 ± 87.61	38.03 ± 37.05	< 0.001
**Poultry, g/d**	5.19 ± 26.35	47.52 ± 48.27	77.02 ± 67.41	< 0.001

All values were adjusted for energy intake using analysis of covariance (ANCOVA)

Values are based on mean ± standard deviation

*P* value less than 0.05 was considered significant

MIND: Mediterranean-DASH diet Intervention for Neurodegenerative Delay; g/d: gram per day

Subjects in the first tertile of MIND had MIND score between (1.5,6.5); second tertile: between (7,9); third tertile: between (9.5–13)

The Multivariate adjusted means for AD according to the tertiles of MIND diet is shown in Table 4. In the crude model, we observed that higher adherence to MIND diet was associated with the lower GAD-7 score (*p* < 0.001). After controlling for covariates these results were remained significant (*p* < 0.001).

**Table 4:** Multivariate adjusted means for GAD-7 score across tertiles of MIND

	**Tertiles of MIND (range)**						
	T1 (1.5,6.5)	T2 (7, 9)	T3 (9.5, 13)	P_1_	P_2_	P_3_	P_4_
GAD-7 score	12.46 ± 3.89	4.31 ± 2.06	4.33 ± 2.06	< 0.001	< 0.001	< 0.001	< 0.001

*P* value less than 0.05 was considered significant

Values are based on mean ± standard deviation

*P* values derived from Analysis of covariance (ANCOVA)

P_1_: crude model

P_2_: adjusted for age, sex, and energy intake

P_3_: additionally, adjusted for marital status, education, medicine use, vitamin supplements use, smoking status, alcohol consumption, physical activity, health status, and past medical history

P_4_: additionally, adjusted for BMI

Subjects in the first tertile of MIND had MIND score between (1.5,6.5); second tertile: between (7, 9); third tertile: between (9.5, 13)

MIND: Mediterranean-DASH diet Intervention for Neurodegenerative Delay

Multivariate adjusted odds ratios and 95% confidence intervals for AD across tertiles of MIND diet score are presented in Table 5. In crude model, those who were in the third tertile of MIND diet score compared to the first tertile were less likely to have AD (OR = 0.04; 95%CI:0.02,0.10). Such a significant association was also seen after taking age, sex, energy intake, marital status, education, medicine use, vitamin supplements use, smoking status, alcohol consumption, physical activity, health status, and past medical history into account. Additional adjustments for BMI revealed a significant reverse association between MIND diet score and odds of AD; such that individuals in the top category of MIND diet score were 97% less likely to have AD compared with those in the bottom category (OR: 0.03, 95% CI: 0.01, 0.09).

**Table 5:** Odds ratios (ORs) and 95% confidence intervals (95% CIs) for Anxiety Disorders according to tertiles of MIND score

	Tertiles of MIND			
	T1	T2	T3	*P* value
**Mean MIND score**	4	8	11	
	OR	OR (95%CI)	OR (95%CI)	
**Crude**	1	0.10 (0.05, 0.22)	0.04 (0.02, 0.10)	< 0.001
**Model I**	1	0.11 (0.05, 0.25)	0.05 (0.02, 0.12)	< 0.001
**Model II**	1	0.07 (0.03, 0.18)	0.03 (0.01, 0.09)	< 0.001
**Model III**	1	0.07 (0.03, 0.19)	0.03 (0.01, 0.09)	< 0.001

Data are OR (95%CI)

Model I: adjusted for age, sex, and energy intake

Model II: additionally, adjusted for marital status, education, medicine use, vitamin supplements use, smoking status, alcohol consumption, physical activity**,** health status, and past medical history

Model III: additionally, adjusted for BMI

MIND: Mediterranean-DASH diet Intervention for Neurodegenerative Delay

We assessed the relationship between MIND diet score with potential predictor variables of AD and demographic variables in Table 6. There was significant reverse linear association between MIND diet score and AD (β = -3.99, *p* < 0.001). Moreover, after controlling for covariates the result remained unchanged (β = -3.63, *p* < 0.001).

**Table 6:** Association between GAD-7 score and MIND

	**MIND**			
	β ± SE	95% CI	aR^2^	*P*_value_
**GAD-7 score**				
Crude	-3.99 ± 0.25	-4.48, -3.49	0.49	< 0.001
Model I	-3.61 ± 0.25	-4.01, -3.12	0.54	< 0.001
Model II	-3.63 ± 0.27	-4.16, -3.09	0.54	< 0.001
Model III	-3.63 ± 0.27	-4.16, -3.09	0.54	< 0.001

*P* value less than 0.05 was considered significant

*β*: unstandardized coefficients

*SE*: standard error

*CI*: confidence interval

*aR*^*2*^: adjusted R square

*P*_value_ obtained from Linear regression

Model I: adjusted for age, sex, and energy intake

Model II: additionally, adjusted for marital status, education, medicine use, vitamin supplements use, smoking status, alcohol consumption, physical activity, health status, and past medical history

Model III: additionally, adjusted for BMI

MIND: Mediterranean-DASH diet Intervention for Neurodegenerative Delay

## Discussion

According to our case–control study, the MIND diet is associated with lower odds of anxiety disorders in adult populations after adjusting for major covariates such that individuals in the highest category of MIND score were 97% less likely to suffer from AD.

Further, the GAD-7 score, demonstrating the severity of anxiety disorders, was negatively correlated with MIND score. There was significant reverse linear association between MIND diet score and AD. As far as we know, it is the first study of its kind investigating the relationship between adherence to MIND diet, the odds, and severity of anxiety disorders.

Our results replicate previous findings that adherence to MIND diet is negatively associated with psychological disorders. In a recent cross-sectional study, it is shown that following MIND diet results in a decrease in odds of depression and anxiety, while no significant association with odds of psychological stress was found [20]. Additionally, results of prospective cohort study using data from Rush Memory and Aging Project (MAP) revealed that adherence to healthy dietary patterns, including DASH, Mediterranean, and MIND diet, plays a protective role against depressive symptoms [37]. In the same way, in a prospective epidemiological investigation an inverse non-linear association between adherence to two established DASH diet indices, called Mellen and the Fung, and depression incidence was explained [38]. Considering Mediterranean diet as another healthy dietary pattern, in a cross-sectional study conducted on 3172 Iranian adults, lower odds of depression, anxiety, and psychological distress was reported in participants in the top tertile of Mediterranean diet [9]. Similarly, in a 12-year prospective study, Hodge et al. reported an inverse association between Mediterranean diet scores and psychological distress [39].

Contrasting our results, in a recently published randomized clinical trial performed by Yau et al. did not find any significant association between anxiety disorders and adherence to the MIND diet in older Chinese people. They mention the small sample, selection bias, and the awareness of participants about the intervention as the limitation of their study [40]. The aforementioned limitations in addition to the various questionnaire used for anxiety assessment, State and Trait Anxiety Inventory (STAI), might have led to such inconsistency. Also, in a cross-sectional study evaluating the association between adherence to the MIND diet, sleep quality, and mental health no significant associations were found between the odds of anxiety, depression, and the MIND diet; however, Greater adherence to this dietary pattern had led to lower odds of poor sleep quality and sleep-related outcomes [41]. The contradictory results can be ascribed to the study population, exclusively males, and the different questionnaires, 21-item depression, anxiety, and stress scale (DASS-21), applied for anxiety assessment. Analyzing the SUN prospective cohort study in 2018, Fersan et al., reported no significant association between adhering to the MIND diet and depression, as well, probably because of the various scoring system adopted in this study [19]. Similarly, in another cross-sectional study anxiety odds were not significantly associated with consumption of the MIND diet, although participants in highest quartile of the MIND diet score had a lower odd of depression and psychological distress [18]. These discordant results might be attributed to the difference in the classification method of the participants, using Hospital Anxiety and Depression Scale (HADS) in this study versus psychiatrist diagnose in our study. According to the randomized controlled trial carried out by Parletta et al. a Mediterranean-style dietary intervention supplemented with fish oil led to a reduction in depression, and improved mental health scores, while this association was not statically significant when anxiety was taken into account [10]. Applying DASS-21 for anxiety assessment, and recruitment of participants based on self-reported depression might be responsible for inconsistent results. Results of another cross-sectional study conducted on 3846 Iranian adults, did not demonstrate any significant relationship between psychological profiles, including depression and anxiety, and adherence to the DASH diet [11]. Such an incompatible result might be ascribed to the different method applied for DASH score calculation, and the different psychological health assessment tool.

A complete understanding of the mechanisms by which the MIND diet may influence brain health remained to be seen. Although, in light of the role of oxidative stress in psychological disorders, it appears that the MIND diet may act as a brain health-protecting agent through its antioxidant and anti-inflammatory properties [42]. MIND diet contains is a great source of vitamins, minerals, and flavonoids playing antioxidant and anti-inflammatory roles leading to brain protection. Furtherly, adherence to MIND dietary patterns means pro-inflammatory food groups constraint including red meat and products, sweets and pastries, butter, and fast foods. Alterations in gut microbiota is another process through which MIND diet impacts brain health. Inflammatory conditions may be triggered by changes in the composition of gastrointestinal microbiota following consumption of pro-inflammatory foods such as fats, sweets, and processed carbohydrates rather than fiber, vegetables, and whole grains [43, 44]. Despite lack of precise predefined mechanism, changes in plasma levels of lipopolysaccharides (LPS) and signaling pathways of inflammasomes, interferon type I, and Nuclear factor kappa B (NF-KB) assumed to be involved [45].

There are several noteworthy strengths in our study that deserved to be highlighted. First of all, previous studies had considered the association between anxiety symptoms and various dietary patterns, but in this study, we considered individuals suffering from anxiety disorders which is a psychological disorder basically different from anxiety symptoms occurrence. Secondly, the classification of the participants was based on psychiatrist diagnosis applying DSM-5 criteria. Furthermore, a validated food frequency questionnaire was used to assess dietary intake, which provided more accurate results compared to other methods. Applying GAD-7, a specifically designed assessment tool for anxiety disorders, to evaluate the severity of AD was another remarkable point.

It is important to acknowledge the limitations of the present study including small number of anxiety cases (*n* = 85) and the case–control design which abstained us from clarifying the temporal relationship. Moreover, the FFQ's dependence on memory may have caused a measurement error. In addition, wine was not considered in the scoring process due to the lack of information in the FFQs. Lastly, we were not able to completely intercept confounding from unmeasured variables, despite adjusting for some potential confounders.

## Conclusion

To sum up, the results obtained in the present case–control study provided some evidence indicating negative association between adherence to MIND diet and the odds of AD. Over and above that, such an inverse relationship exists between the severity of AD, evaluated by GAD-7, and the MIND score. Finally, due to the probable preventive role of diet, it is vital to clarify the association between diet and psychological disorders through large-scale prospective cohort studies in the future.

## Data Availability

The datasets generated or analyzed during the current study are not publicly available due to restrictions e.g. their containing information that could compromise the privacy of research participants but are available from the corresponding author on reasonable request.
